# LI-RADS v2017 for liver nodules: how we read and report

**DOI:** 10.1186/s40644-018-0149-5

**Published:** 2018-04-24

**Authors:** Wolfgang Schima, Jay Heiken

**Affiliations:** 1 Department of Diagnostic and Interventional Radiology, Goettlicher Heiland Krankenhaus, Barmherzige Schwestern Krankenhaus, and St. Josef Krankenhaus, Vienna, Austria; 2 Mayo Clinic, Rochester, Minnesota USA

**Keywords:** Hepatocellular carcinoma, CT, MRI, LI-RADS

## Abstract

The Liver Imaging Reporting and Data System (LI-RADS) standardizes the interpretation and reporting of imaging examinations in patients at risk for hepatocellular carcinoma (HCC). For focal liver observations it assigns categories (LR-1 to 5, LR-M, LR-TIV), which reflect the relative probability of benignity or malignancy of the respective observation. The categories assigned are based on major and ancillary image features, which have been developed by the American College of Radiology (ACR) and validated in many studies. This review summarizes the relevant CT and MRI features and presents an image-guided approach for readers not familiar with LI-RADS on how to use the system. The widespread adoption of LI-RADS for reporting would help reduce inter-reader variability and improve communication among radiologists, hepatologists, hepatic surgeons and oncologists, thus leading to improved patient management.

## Background

Recent years have seen enormous advances in the multi-modality treatment of hepatocellular carcinoma (HCC), which have brought substantial improvement in prognosis of HCC patients. Thus, early detection of liver nodules, accurate diagnosis of HCC and tumour staging for treatment planning have become increasingly important. Several societies (including the American Association for the Study of Liver Diseases [AASLD], the European Association for the Study of the Liver [EASL], the Japan Society of Hepatology [JSH], and others) have developed guidelines for utilization of imaging tests for the diagnosis of HCC [1–3].

These guidelines rely on a few criteria, including size, arterial phase hyperenhancement, washout, a certain level of nodule growth on serial examinations and/or histology for diagnosis of HCC. Although these criteria are helpful in making the diagnosis of HCC in certain patients, they do not cover the broad spectrum of imaging findings, which may be encountered in patients with chronic liver disease and focal liver nodules. Thus, the American College of Radiology (ACR) convened a panel of expert radiologists to develop a new and comprehensive system for interpretation and reporting CT and MRI examinations of the liver in patients at risk for HCC. LI-RADS (Liver Imaging Reporting and Data System) was launched in 2011, with recent updates in 2014 and 2017 [4]. It is important to be familiar with this system to categorize reliably lesions in patients with chronic liver disease [5, 6].

Several reports have tried to provide a comprehensive overview of the LI-RADS system and an introduction into the use of the system [7–10]. The present manuscript focuses on the application of LI-RADS in clinical practice by using a step-by-step approach, illustrated by multiple case examples.

## Which patient population?

The LI-RADS classification system should be applied only to patients with cirrhosis or chronic hepatitis B infection or with current or prior HCC. Is should not be applied to patients under the age of 18 years, or to patients with cirrhosis due to special conditions (congenital hepatic fibrosis or due to vascular disorders, such as Budd-Chiari syndrome, cardiac congestion or diffuse nodular regenerative hyperplasia).

## How to do the imaging

For MDCT (with at least an 8-row scanner) a triple-phasic contrast enhanced study is recommended, comprising a late arterial, portal venous, and delayed phase. An unenhanced scan is required in patients with previous loco-regional tumour treatment. No specific recommendations are given for administration of contrast material, scan delay, slice thickness, reconstruction interval, or other image acquisition and display parameters. However, many excellent papers about optimization of CT protocols in patients with chronic liver disease have been published [11–17].

For MR imaging, either 1.5 T or 3.0 T units may be used with a torso phased-array coil. MR protocol has to include unenhanced T1w in- and opposed-phase, T2w turbo spin echo (TSE), preferably with fat saturation, and multi-phasic contrast-enhanced T1w imaging in the late arterial, portal venous and delayed phases after IV administration of non-specific gadolinium chelates. After administration of the liver-specific MR contrast agent gadoxetate disodium (Primovist® or Eovist®, Bayer Healthcare, Germany) or gadobenate dimeglumine (MultiHance®, Bracco, Italy) hepatobiliary phase images are acquired. Use of diffusion-weighted pulse sequences is suggested.

## How shall I rate nodules according to LI-RADS?

**Fig. 1:**
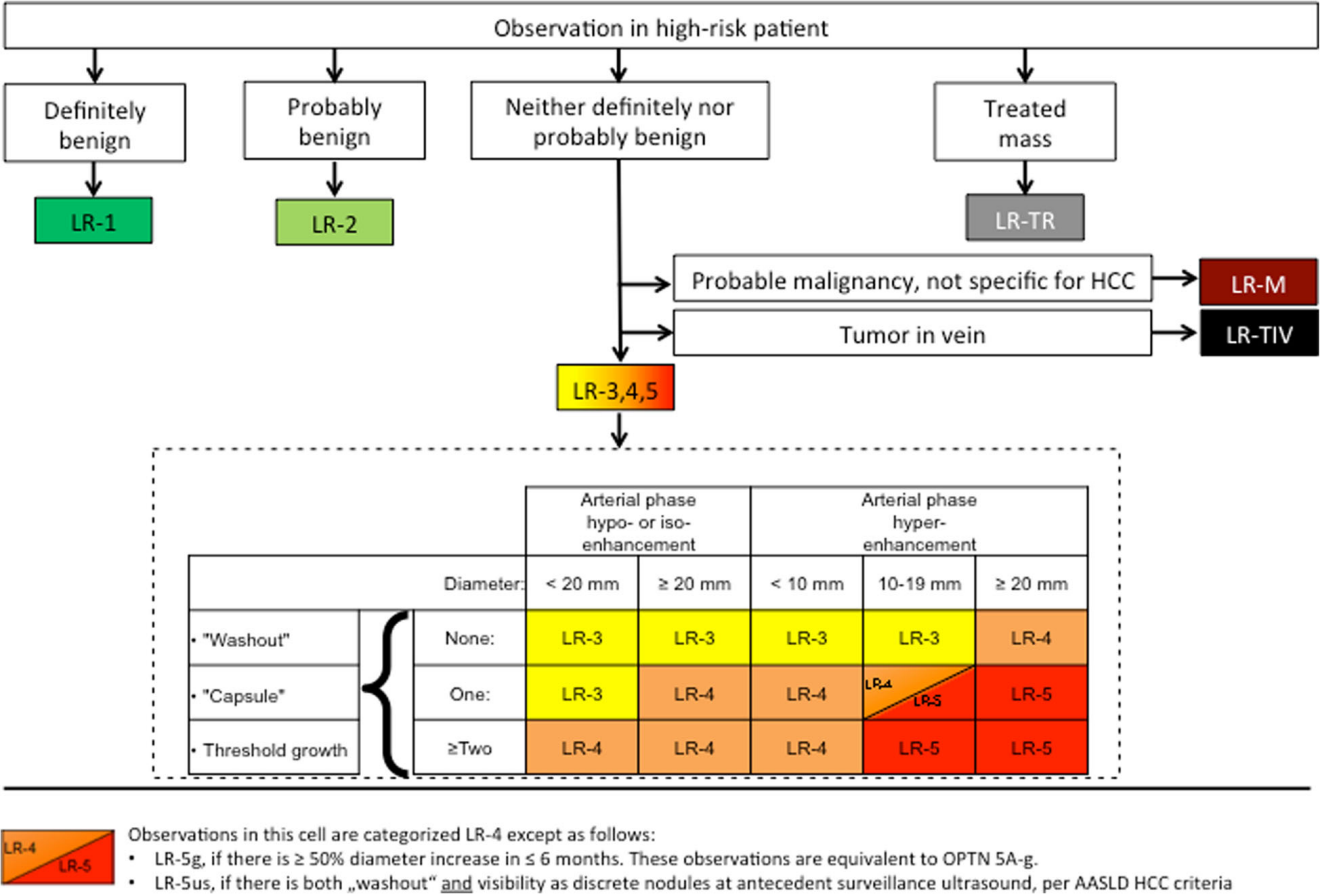
LI-RADS Diagnostic Algorithm

The LI-RADS category is a score assigned to a focal liver observation. The categories encompass the spectrum of benign to malignant observations encountered in patients with chronic liver disease at risk for cirrhosis. It does not apply to patients without chronic liver disease. LI-RADS observations are categorized according to imaging features and/or growth as LI-RADS 1 to 5 (from definitely benign to definitely HCC) (Table 1, Fig. 1). The diagnostic algorithm uses major features to categorize LR-3, LR-4, and LR-5 observations. In addition there are ancillary features, which can be used to adjust the preliminary LI-RADS category. Some ancillary features favour benignity whereas others favour malignancy. The presence of these features can be used to decrease or increase the category (down to LR-1 or up to LR-4), but not up to LR-5.

**Table 1:** LI-RADS Categories of Nodules

Category	Judgement	Rationale
LR-1	Definitely benign	100% certainty the observation is benign
LR-2	Probably benign	High probability the observation is benign
LR-3	Intermediate probability for HCC	Both HCC and benign entity have moderate probability. Observation does not meet criteria for other LR category
LR-4	Probably HCC	High probability the observation is HCC, but no 100% certainty
LR-5	Definitely HCC	100% certainty the observation is HCC
LR-M	Probably malignant, not specific for HCC	Observation is probably malignant, but imaging features not specific for HCC (suggestive of non-HCC malignancy)
LR-TIV	Definitely tumour in vein	Unequivocal enhancing soft-tissue tumour in vein. Visualization of a parenchymal mass is not required.
LR-TR	Treated observation	Any observation, which has undergone loco-regional treatment
LR-NC	Not characterizable	Observation cannot be characterized due to image degradation or omission of scans/pulse sequences

The steps to assess an observation are as follows: (1) apply the LI-RADS Diagnostic Algorithm (Fig. 1). (2) Apply the major criteria for all observations not categorized as LR-1, LR-2, LR-M or LR-TIV. (3) Apply ancillary features favouring either malignancy or benignity. (4) Apply tie-breaking rule: if there is uncertainty about the category to be chosen, then choose the category with less certainty (e.g. if unsure about LR5 or LR4, then choose LR4). (5) During the final check the radiologist has to question whether the provisionally assigned category is reasonable.

### Step 1: The diagnostic algorithm (Fig. 1)

The Diagnostic Algorithm is used to assign categories LR-1 and LR-2 to observations that are definitely or probably benign. If definite tumour is depicted in a vein, then LR-TIV is assigned. LR-M is given if the morphology and enhancement characteristics suggest a non-HCC malignancy. If the imaging study does not allow adequate assessment of major and ancillary features due to image degradation or omission of important scans/sequences, then LR-NC is assigned.

### Step 2: Major criteria

**Fig. 2:**
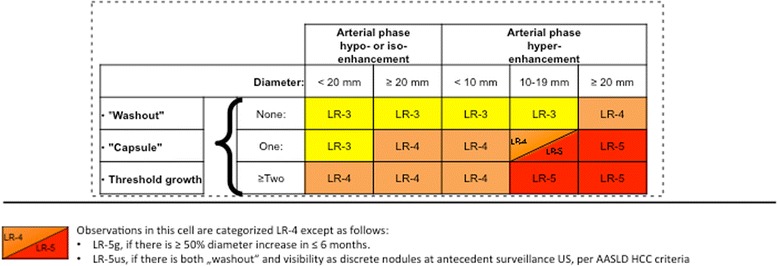
Application of major criteria for assigning LI-RADS categories LR-3 – LR-5

**Fig. 3:**
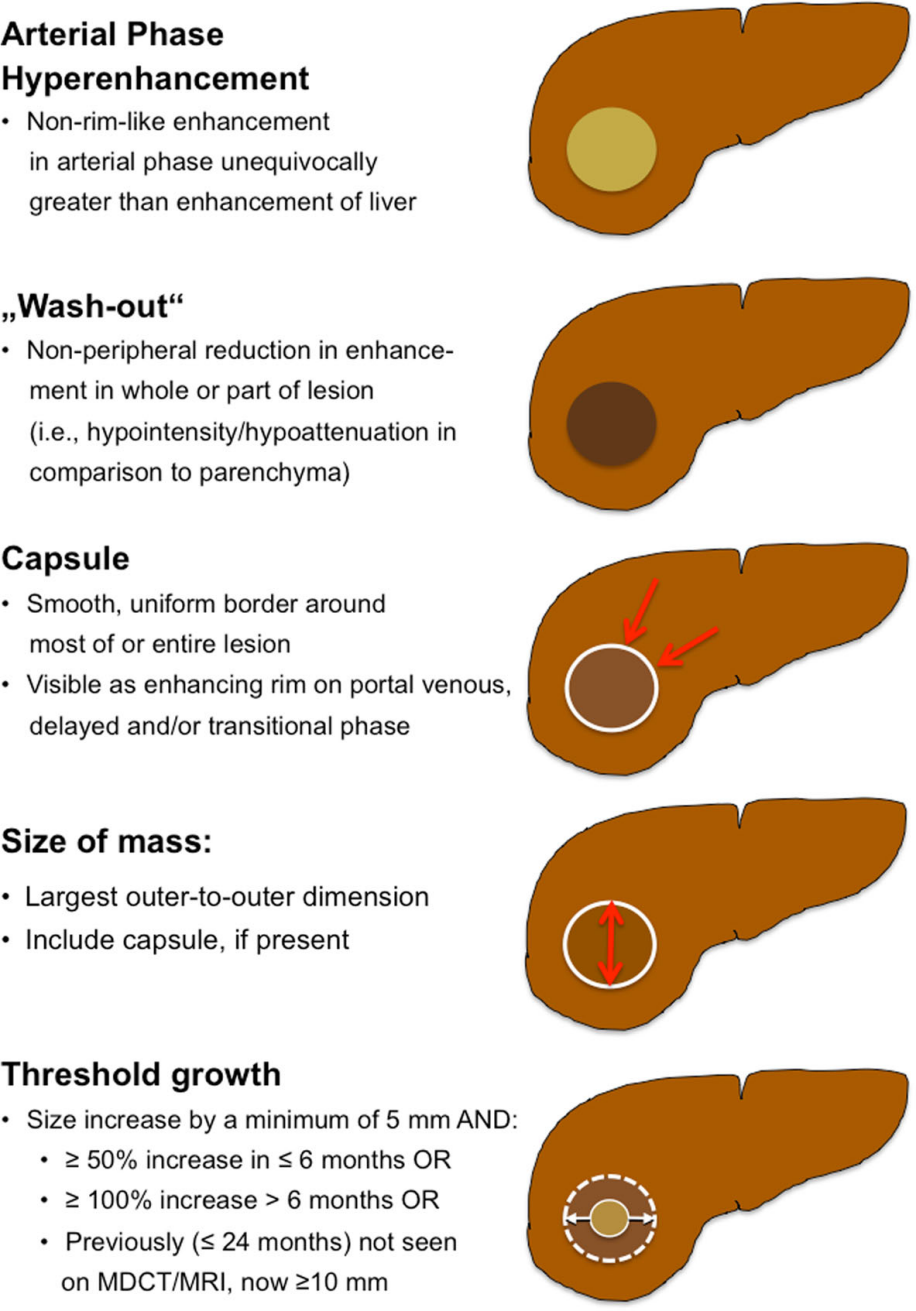
LI-RADS major imaging features

For all other observations, the major criteria are applied for evaluation of liver nodules: observation size (< 10 mm, 10–19 mm, ≥ 20 mm), arterial phase hyper-enhancement or arterial phase hypo- or iso-enhancement, washout appearance (signal intensity/attenuation loss to hypointensity/hypoattenuation in the venous and/or delayed phase), presence of an enhancing “capsule” around the lesion and threshold growth (minimum increase of 5 mm and ≥ 50% diameter increase in ≤6 months or ≥ 100% increase in ≤1 year). These major features are used to assign an observation to categories LI-RADS 3–5 (Figs. 2, 3). Each observation is assigned a LI-RADS category according to the major features observed (Fig. 3).

#### Definition of major imaging features that favour the diagnosis of HCC

1. ***Arterial Phase Hyperenhancement:***

At least part of the observation or the entire observation must show enhancement greater than the surrounding liver parenchyma during arterial phase imaging (Fig. 3). This imaging feature is the single most important feature in patients with HCC, reflecting new angiogenesis in a developing HCC. It is important to note that hyperenhancement has to be non-rim-like (which would favour the diagnosis of a non-HCC malignancy).

2. ***Portal Venous Phase or Delayed Phase Hypoenhancement (Washout):***

Hypoenhancement of an observation in the venous phase and/or the delayed phase (so-called washout) is also seen as a strong predictive factor of HCC, especially in combination with arterial phase hyperenhancement. Be aware that the definition of washout requires a temporal reduction of nodule enhancement relative to surrounding liver parenchyma from an earlier to a later phase (Fig. 3). Hypoattenuation/hypointensity of a nodule in a single enhanced venous phase scan (without demonstration of arterial phase hyperenhancement) does not fulfil the definition of washout. Washout must comprise the observation in whole or in part, but not in a rim-like pattern. The presence of arterial phase hyperenhancement and venous and/or delayed phase washout is not a sensitive, but a specific sign for HCC diagnosis in the at risk population. Particular attention should be paid to co-locating the observation on arterial phase, venous and/or delayed phase images to make sure that hyper- and hypoenhancement occur at the exact same location.

3. ***Capsule Appearance:***

An enhancing “capsule” is a major feature. Although the term “capsule” is used in the literature, the distinction between a true tumour capsule and a (non-tumorous) pseudocapsule cannot be made by imaging alone [18], but that differentiation is not necessary for imaging [19]. Capsule appearance is defined as a smooth, thin enhancing rim of tissue around an observation (Fig. 3). It may be seen in the portal venous, the delayed and/or the transitional (if liver-specific MR contrast agent was administered) phase. It must not be confused with a perfusion abnormality around an observation, which is less distinct. An enhancing “capsule” tends to show progressive enhancement from early to later phases, in contrast to a perfusion abnormality, which usually fades with time. A non-enhancing “capsule” is categorized only as an ancillary feature favouring malignancy. Usually it is seen on T2-weighted or hepato-biliary phase images (Table 2). Reader perception of an enhancing “capsule” influences the interpretation of nodule washout: in nodules with enhancing “capsule”, readers were more likely to subjectively rate washout to be present than it would have been based on objective criteria [20]. In general, “capsule” appearance has a slightly higher sensitivity, but similar specificity to washout appearance for diagnosis of HCC [21].

4. ***Size:***

**Table 2:** Ancillary features favouring either malignancy or benignity

Ancillary features favouring malignancy in general	Ancillary features favouring benignity
• US visibility as discrete nodule	• Size stability > 2 years
• Subthreshold growth	• Size reduction
• Restricted diffusion	• Enhancement parallels blood pool
• Mild to moderate T2 hyperintensity	• Undistorted vessels
• Corona enhancement	• Iron in mass (more than liver)
• Fat sparing in solid mass	• Marked T2 hyperintensity
• Iron sparing in solid mass	• Hepato-biliary phase isointensity
• Transitional phase hypointensity	
• Hepato-biliary phase hypointensity	
Favouring HCC in particular	
• Non-enhancing “capsule”	
• Nodule-in-nodule	
• Mosaic architecture	
• Blood products in mass	
• Fat in mass, more than adjacent liver	

Size of an observation should be measured in the largest outer-to-outer dimension. Often arterial phase images show perilesional perfusion alterations, such as arterio-portal shunting or corona enhancement, which can make the lesion appear larger than it is in reality. Thus, measurement should not be taken in the contrast-enhanced arterial phase or on DWI (with similar problems of blurred margins being quite prevalent), if margins are well visualized on other images.

5. ***Threshold Growth:***

Interval growth of an observation is highly predictive of HCC (or other malignancies) and is defined by 3 different scenarios. First, threshold growth is fulfilled by growth of an observation of at least 50% in longest dimension in ≤6 months, with a minimum size increase of 5 mm. This feature is important for characterisation of lesions that are small at the baseline scan. Second, if the follow-up study is performed later than at 6 months, then a 100% increase in lesion size is required. Third, a lesion not seen on previous MDCT or MRI (obtained up to 24 months before the study) that has now grown to a size of at least 10 mm.

### Step 3: Ancillary features

In clinical practice, the presence of one or more ancillary features at MDCT/MRI would make us lean subjectively toward diagnosing an observation as either benign or malignant [22]. With LI-RADS a more formal approach is taken. Ancillary features favouring HCC diagnosis include the following (Table 2): hepatobiliary phase hypointensity (after administration of liver-specific MR contrast agent), transitional phase hypointensity, mild to moderate T2 hyperintensity, restricted diffusion, distinctive rim, corona enhancement, mosaic architecture, nodule-in-nodule architecture, intra-lesional fat, lesional iron or fat sparing, blood products, and diameter increase less than the threshold growth. The use of hepato-biliary MR contrast agents has been shown to be helpful, because contrast enhancement characteristics of an observation in the hepato-biliary phase may rule in or rule out certain entities [23]. The presence of ancillary features favouring malignancy may be used to upgrade by one category, but not beyond LR-4 (e.g. from LR-3 to LR-4). Absence of ancillary features must not be used to downgrade an LR category. Ancillary features that favour benign histology (Table 2) can be used to downgrade an observation by one category (e. g., from LR-4 to LR-3 or from LR-3 to LR-2).

### Steps 4 and 5: Tie-breaking rule and final check

If unsure between two categories during assessment of an observation, then choose the category with lower certainty. This means that LR-2 (probably benign) instead of LR-1 (definitely benign) or LR-4 (probably HCC) instead of LR-5 (definitely HCC) should be reported. If unsure, whether HCC or a non-HCC malignancy is present, then LR-M should be assigned (lower certainty of hepatocellular origin), which would prompt biopsy.

During the final check, the reader has to ask him/herself, if the assigned category is reasonable and appropriate. If not, then reassessment of the observation is necessary.

## LI-RADS categories

### LR-1

**Fig. 4:**
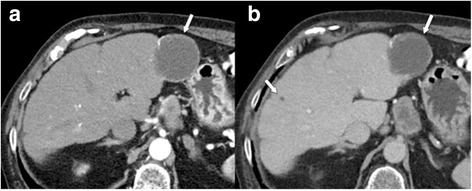
LR-1 **a** Arterial and **b** venous phase MDCT images show a large, calcified cyst in the left lateral segment and a smaller definitely cystic lesion in the right lobe (only seen on the venous phase image). LR-1 (definitely benign)

An LR-1 observation is considered definitely benign. Examples of LR-1 include definitive cyst, haemangioma, focal fat accumulation or sparing, confluent fibrosis, etc. (Fig. 4). The diagnosis can be made according to unequivocal appearance or knowledge of prior studies.

### LR-2

**Fig. 5:**
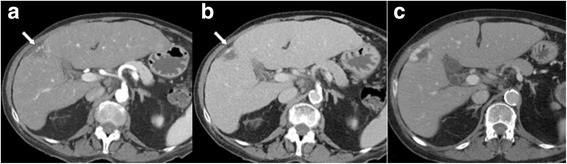
LR-2 **a** Arterial phase MDCT shows a subcapsular lesion in the right lobe with capsular retraction and peripheral nodular enhancement, with **b** progressive centripetal enhancement in the venous phase. Diagnosis is probable haemangioma (LR-2). **c** A previous CT scan, performed 9 years earlier, could be obtained, which confirmed the diagnosis of haemangioma

**Fig. 6:**
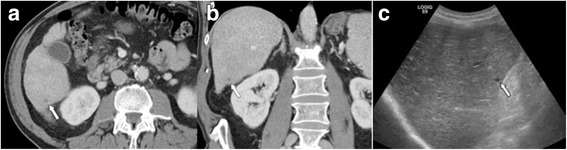
LR-2. Venous phase MDCT in the **a** axial and **b** coronal plane shows a small indistinct hypoattenuating observation in the right lobe (arrows). No enhancement was seen in other phases. Diagnosis of probable cyst (LR-2). **c** Ultrasound was performed, which confirmed the presence of a small subcapsular cyst (arrow)

Probably (but not definitely) benign observations are categorized as LR-2 (Figs. 5, 6). In addition to the diagnoses mentioned above (if not made with 100% certainty), typical perfusion abnormalities (transient hepatic attenuation differences, THAD) are categorized as LR-2. Circumscript hepatocellular nodules without any suspicious feature would also fall in this category. In the study of Tanabe et al. [24] all LR-2 observations (made at CT or MRI) remained stable or decreased in category.

### LR-3 – LR-5

**Fig. 7:**
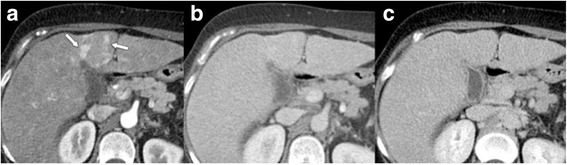
LR-3. **a** Arterial phase MDCT shows 2 intraparenchymal observations of 1.6 and 1.3 cm in S4 (arrows), which are hypervascular, but do not show washout in **b** venous or **c** delayed phases. According to size (10–19 mm), presence of arterial phase hypervascularity, and absence of other major features, lesions were categorized as LR-3. Diagnosis of transient hepatic attenuation difference is most likely. No HCC was found during follow-up

**Fig. 8:**
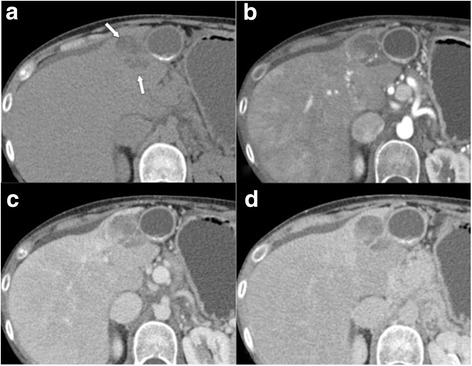
LR-4. **a** Unenhanced MDCT shows a 3.5 cm mass adjacent to the gallbladder (arrows). Measured density is − 9 HU, indicative of intralesional fat. Enhanced images in the **b** arterial, **c** venous, and **d** delayed phases show moderate marginal enhancement (no arterial phase hypervascularity) without washout. Accordingly, observation would be categorized LR-3, but presence of intralesional fat indicates upgrade to LR-4. Lesion was biopsied, which revealed a fatty regenerative nodule

**Fig. 9:**
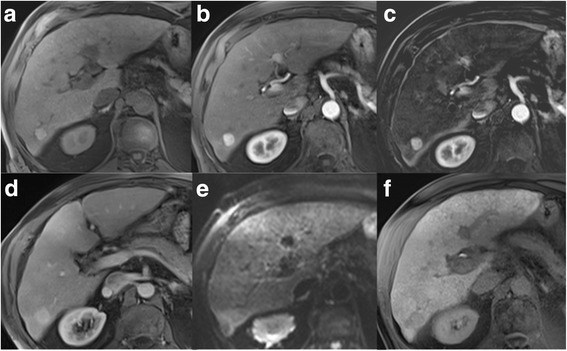
LR4. **a** Unenhanced T1w GRE image reveals a 17 mm nodule in the right lobe, which is **b** hypervascular in the arterial phase, best seen **c** in the subtraction image. **d** There is no washout present in the venous phase. **e** Ancillary features favouring malignancy are restricted diffusion in DWI and **f** hypointensity in hepatobiliary phase. According to size and hypervascularity without washout, lesion would be categorized as LR-3, but ancillary features justify upgrade to LR-4. Biopsy revealed HCC

**Fig. 10:**
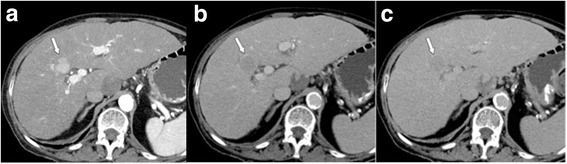
LR-5. **a** Arterial phase MDCT demonstrates a 23 mm hypervascular nodule adjacent to the right portal vein (arrow), which shows washout in both **b** venous and **c** delayed phases (arrows). In addition, a thin enhancing “capsule” is seen on the portal venous phase image. Lesion was categorized as LR-5 (HCC)

**Fig. 11:**
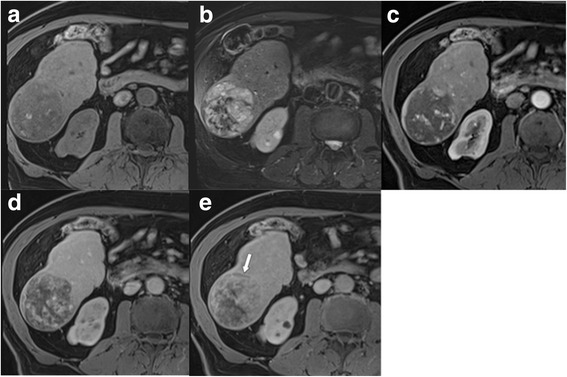
LR-5 **a** Encapsulated mass in the right lobe, which is **a** hypointense on T1 with some bright spots (indicative of haemorrhage), **b** moderately hyperintense on T2, and shows **c** inhomogenous hypervascularity in the arterial phase, but no washout in **d** venous and **e** delayed phases. According to size (≥ 20 mm), hypervascularity without washout, and enhancing “capsule” (best seen in delayed phase, arrow) mass is categorized as LR-5. Ancillary findings favouring HCC are intralesional blood products

The major criteria (Fig. 3) of size (< 10 mm, 10–19 mm, ≥20 mm), arterial phase hyperenhancement, washout in the portal venous and/or delayed phase, enhancing “capsule”, and threshold growth determine the LI-RADS category of observations not suited for the benign classes LR-1, LR-2 (Figs. 7, 8, 9, 10 and 11). After provisional categorization according to these criteria, application of ancillary features can alter final judgement by one category down or up (but not up to LR-5) (Fig. 9). The most common cause of LR-3 observation is a hypervascular pseudolesion [25]. During follow-up of LR-3 lesions, 80% remained stable and 14% decreased in size or were no longer visible [25]. Only 6% of LR-3 turned out to be probable or definite HCC. In another study, 4% and 5% of LR-3 progressed to LR-4 or LR-5, respectively [24]. This allows the conclusion that follow-up imaging at 3–6 months (with the same or an alternative modality) is an appropriate strategy. In two recent studies, 31–38% of LR-4 observations progressed to a definitely malignant category during follow-up [24, 26]. For an LR-4 or LR-5 observation (Figs. 8, 9, 10 and 11) a multidisciplinary discussion for consensus management is needed [4].

If the diagnosis of an HCC is made (LR-4 or LR-5), then a careful search for the presence of tumour in the vein is warranted, because this would move the observation into the category LR-TIV.

### LR-TIV (definitely malignant with tumour in vein)

**Fig. 12:**
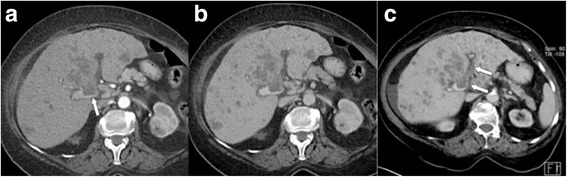
LR-TIV. **a** Arterial phase MDCT shows a large hypovascular mass, which invades the portal vein bifurcation (arrow). There are multiple solid nodules present in both lobes, most likely metastases. **b** Venous phase MDCT confirms hypovascularity of the tumour, which was subsequently proven to be CCC. **c** Paraxial volume-rendered technique demonstrates the extent of tumour thrombosis in the left system (arrows) and the presence of cirrhosis with ascites

Definite presence of tumour within a vein is indicated by unequivocal enhancing soft tissue present in the vessel lumen, no matter if a definitive parenchymal mass is seen. Additional features include vein occlusion with ill-defined walls, expansion of the lumen, restricted diffusion in the vein on DWI, and an occluded vein being in contiguity with a malignant parenchymal mass [27–29] (Fig. 12). Pitfalls in diagnosing tumour in a venous lumen may occur, if early venous enhancement due to arterial portal shunting is mistaken as enhancing tumour. Likewise, in late arterial phase MDCT imaging (which is the preferred phase for detection of hypervascular HCC) early enhancement of the portal vein is routinely seen. It is of great importance to differentiate between HCC and other non-HCC malignancies because of different treatment strategies. In the previous LI-RADS edition, the observation of the presence of a tumour in vein (whether or not an intraparenchymal HCC is visible) was assigned to LR-5 V category. However, a recent study [30] has shown that portal vein tumour can also be observed in non-HCC malignancies (LR-5 M category), such as intrahepatic cholangiocarcinoma, combined hepatocellular-cholangiocarcinoma or metastases. Thus, the category of LR-TIV was created, which comprises all observations with tumour in a vein. The most likely aetiology of the venous tumour invasion should be indicated in the radiology report.

### LR-M

**Fig. 13:**
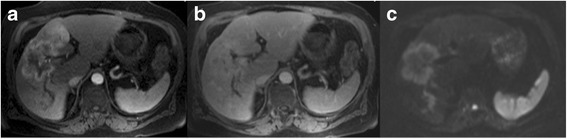
LR-M **a** Arterial phase MRI showing peripheral hyperenhancement. **b** Delayed phase image (at 5 min) showing central enhancement. **c** At DWI a targetoid appearance is apparent. Image features are not in line with HCC, but indicative of LR-M. Lesion was proven to be CCC

The imaging criteria for an observation to be included in the category LR-M (probably or definite malignancy, but not specific for HCC) have been redefined in the LI-RADS v2017. The central criteria are a target-like morphology, which likely reflects peripheral tumour hypercellularity (showing rim enhancement) and central stromal fibrosis (which may show progressive enhancement in the delayed phase). This imaging appearance is characteristic (but not pathognomonic) of cholangiocarcinoma (CCC) and hepatocholangiocarcinoma, but can also be seen in other malignancies (such as metastases, etc.). Targetoid enhancement features include rim-like arterial phase enhancement with peripheral washout as well as delayed phase progressive enhancement of the tumour centre, which is shown in 42–96% of lesions [21] (Fig. 13). Targetoid appearance also can be seen on DWI or hepato-biliary phase images.

Category LR-M also comprises lesions with non-targetoid appearance, which are suspicious for malignancy, but not typical for HCC: infiltrative growth, marked diffusion restriction, necrosis, liver surface retraction, and biliary obstruction to a higher degree than expected from the size of the mass. In hepatocholangiocarcinoma (biphenotypic liver cancer), 54% of lesions meet the criteria for HCC, if only the LI-RADS major features are considered. However, 88% of those show at least one ancillary feature favouring non-HCC malignancy [17], which underscores the importance of ancillary features for appropriate classification.

If the contrast enhancement characteristics and morphology of a lesion are clearly suspicious for malignancy, but the diagnosis of HCC cannot be made with 100% certainty, then according to the tie-breaking rules the category with lower certainty (LR-M) should be chosen.

### LR-TR

The treatment response (TR) categories are used to assess tumour response after loco-regional therapy. Post-treatment imaging is preferably performed with the same imaging modality at 3-month intervals. In many institutions, the first post-treatment study is acquired at 1 month after therapy to have a baseline.

Treatment response categories comprise LR-TR nonviable, viable, equivocal, and nonevaluable, depending on treatment effect and the certainty, with which the treated lesion can be assessed. Features indicative of viable tumour include: nodular, mass-like or thick irregular rim enhancement of the treated lesion, plus: arterial phase hyperenhancement, washout or enhancement similar to the pre-treatment phase.

**Fig. 14:**
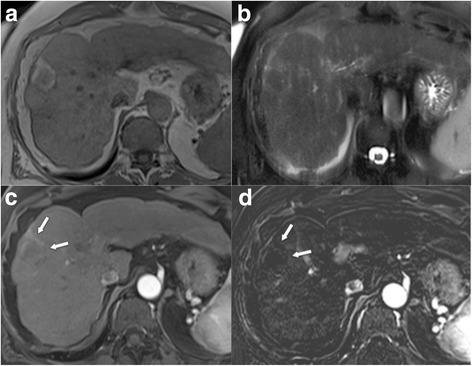
LR-TR nonviable **a** T1w GRE image after tumour ablation demonstrates hyperintense necrosis zone, which is **b** hypointense on T2w TSE image. **c** Contrast-enhanced arterial phase images shows absence of lesion enhancement (arrows). **d** However, this best seen on the subtracted arterial phase image (arrows)

Absence of lesion enhancement and expected treatment-specific enhancement are suggestive of LR-TR nonviable (Fig. 14). However, readers have to be aware of the fact that radiologic sign of nonviability is not synonymous with pathologic complete response, because imaging is not sensitive to small residual tumour foci.

## Conclusion

In conclusion, LI-RADS is a diagnostic system developed by the ACR to standardize terminology, interpretation, and reporting of liver studies in patients at risk for HCC. The widespread adoption of LI-RADS for reporting would help to reduce inter-reader variability and, thus, produce more consistent diagnoses. Updates, which take into account the evolving scientific evidence, will help to improve not only diagnostic performance, but also patient management.
